# The association between dietary inflammatory index and latent tuberculosis infection: Confounding role of country of birth

**DOI:** 10.1016/j.clinsp.2026.100937

**Published:** 2026-04-03

**Authors:** Jingbo Jia, Yuanyuan Liu, Hua Zhang, Jianrui Pi, Chao Wang, Shiqi Dong, Tong Zhang, Wanjie Yang

**Affiliations:** a Tuberculosis Department of Tianjin Haihe Hospital, Tianjin, China; b TCM Key Research Laboratory for Infectious Disease Prevention for State Administration of Traditional Chinese Medicine, Tianjin, China; c Nursing Department of Tianjin Haihe Hospital, Tianjin, China; d The Blood Purification Center of Tianjin Haihe Hospital, Tianjin, China; e The President's Office of Tianjin Haihe Hospital, Tianjin, China

**Keywords:** Dietary inflammatory index, DII, Tuberculosis, LTBI, NHANES

## Abstract

**Background:**

Mounting evidence suggests that diet-induced inflammation may modulate the risk of Latent Tuberculosis Infection (LTBI). However, the reported association between the Dietary Inflammatory Index (DII) and LTBI remains inconsistent, and potential confounding factors such as country of birth have not been fully elucidated in previous studies.

**Objective:**

This study aimed to evaluate the confounding effect of country of birth on the apparent association between DII and LTBI, using data from the National Health and Nutrition Examination Survey (NHANES). A key hypothesis was tested that the observed relationship between DII and LTBI could be explained by the stratification of nativity status.

**Methods:**

A cross-sectional analysis was conducted on 3892 eligible participants from NHANES with no missing values for core variables. Comprehensive variables were collected, including core indicators DII,LTBI status, and active tuberculosis status defined in accordance with the diagnostic criteria specified in the NHANES operational guidelines), demographic characteristics (country of birth, age, gender, ethnicity, household size), socioeconomic factors (educational attainment, Poverty Income Ratio [PIR], marital status), and health-related covariates (Body Mass Index [BMI], smoking habits, alcohol intake, Hypertension [HPT], Diabetes Mellitus [DM]). Logistic regression models, smooth curve fitting, and stratified analyses by country of birth (US-born vs. non-US-born) were applied to quantify the association between DII and LTBI, with sequential adjustment for potential confounders.

**Results:**

The DII values of the participants ranged from -4.63 (highly anti-inflammatory diet) to 5.47 (highly pro-inflammatory diet), with a mean of 1.40±1.91, indicating a slight pro-inflammatory dietary tendency overall. LTBI prevalence varied by TB classification, and TB-positive groups showed significantly higher active tuberculosis rates than the TB-negative group. In the initial multivariate logistic regression model, adjusted for demographic, socioeconomic, and health-related factors (excluding country of birth), a significant inverse association was observed between DII and LTBI (OR = 0.93, 95 % CI 0.88–0.98, *p* = 0.011). However, this association was attenuated to non-significance after further adjustment for country of birth (OR = 0.96, 95 % CI 0.90–1.01). Stratified analyses confirmed the lack of a significant association between DII and LTBI in both US-born (OR = 0.95, 95 % CI 0.88–1.04) and non-US-born (OR = 0.96, 95 % CI 0.88–1.03) subgroups, with all interaction p-values > 0.05. Additionally, the initial linear dose-response relationship between DII and LTBI was no longer maintained after controlling for country of birth. The authors acknowledge that the country of birth variable is a relatively crude indicator, and residual confounding may persist in the current analysis.

**Conclusions:**

In this nationally representative sample, the initially observed inverse association between DII and LTBI was not sustained after adjusting for country of birth, indicating that nativity status is a critical confounder of the apparent DII-LTBI relationship. The findings highlight that the previously reported protective effect of an anti-inflammatory diet against LTBI may be attributable to the confounding influence of country of birth rather than a direct causal effect. Future epidemiological studies investigating diet-infection relationships should rigorously assess and control for country of birth as a potential confounding factor, and explore more refined indicators to reduce residual confounding.

## Highlights

• Initial multivariate analysis showed inverse DII-LTBI association (OR = 0.93, *p* = 0.011).
• Association became non-significant after adjusting for country of birth (OR = 0.96).
• No significant link found in US-born or non-US-born subgroup analyses.
• Country of birth is identified as a key confounder in the diet-LTBI relationship.

## Introduction

The global repercussions of Tuberculosis (TB) persist as a substantial public health dilemma,^1^, ^2^, ^3^ with Latent Tuberculosis Infection (LTBI) acting as a vital reservoir for prospective future cases of active TB.^4^^,^^5^ LTBI is defined by the existence of Mycobacterium tuberculosis in individuals who exhibit no clinical manifestations and are incapable of spreading the infection to others. The World Health Organization (WHO) estimates that approximately one-quarter of the world's population is afflicted with LTBI, which markedly amplifies the likelihood of reactivation into active TB disease, particularly among vulnerable populations.^6^ This unnoticed epidemic underscores the pressing demand for effective indicators that facilitate early detection to avert the transition from LTBI to active TB, thereby alleviating the related healthcare burden.^7^, ^8^, ^9^

Recent studies have begun to explore the relationship between diet, inflammation, and the immune response. Diet and individual nutrients can influence systemic markers of immune function and inflammation,^10^, ^11^, ^12^ and may also influence people’s TB-related health outcomes. A growing body of evidence suggests that diet can significantly affect immune responses, potentially altering the course of chronic diseases. For instance, diets high in processed foods and sugars are associated with increased systemic inflammation, which may impair the immune system's ability to combat chronic diseases.^13^ Conversely, diets rich in fruits, vegetables, and whole grains have been associated with lower levels of inflammatory markers, suggesting a protective effect against chronic inflammatory conditions and possibly enhancing immune function.^14^

The DII is a tool developed to assess the inflammatory potential of an individual's diet. However, the association between DII and LTBI remains unclear. A critical, yet often overlooked, potential source of confounding by nativity status (country of birth). The country of birth can, to some extent, measure various early life and environmental exposure factors, including TB endemicity, socioeconomic status, and cultural dietary habits, all of which could independently influence both diet and LTBI risk. Therefore, utilizing a large, nationally representative sample from the National Health and Nutrition Examination Survey (NHANES), this study aimed to investigate the hypothesis that the apparent association between DII and LTBI is confounded by country of birth. The present analysis sought to clarify this relationship by systematically adjusting for nativity status and other potential confounders.

## Materials and methods

### Data and eligibility criteria

This cross-sectional analysis was conducted using data from the 2011–2012 National Health and Nutrition Examination Survey (NHANES), a program administered by the Centers for Disease Control and Prevention (CDC) .^15^ The core objective of NHANES is to evaluate the health and nutritional status of non-institutionalized U.S. residents via a rigorous multistage probability sampling approach.^16^ NHANES amasses comprehensive demographic, socioeconomic, and health-related data through in-home interviews, standardized health examinations, and laboratory assays conducted at Mobile Examination Centers (MECs).

The study protocol was reviewed and approved by the Ethics Review Committee of the National Center for Health Statistics (NCHS). All participants provided written informed consent prior to enrollment, and secondary analysis of de-identified NHANES data did not require additional institutional review board approval.^17^ NHANES data are publicly accessible via the official website (https://wwwn.cdc.gov/nchs/nhanes/default.aspx), with data extraction completed on November 26, 2024.

Eligibility criteria for the present study included participants who completed Tuberculosis (TB) testing and had complete data on the Dietary Inflammatory Index (DII), Latent Tuberculosis Infection (LTBI) status, country of birth, and all predefined covariates. Participants with missing values for any of these key variables were excluded from the final analysis. This study was reported in adherence to the STROBE Statement guidelines for cross-sectional research.

### Definition of LTBI

LTBI status was ascertained using two validated diagnostic assays available in the 2011–2012 NHANES dataset: the Tuberculin Skin Test (TST) and the QuantiFERON® TB Gold In-Tube (IGRA) assay. A TST result was classified as positive if the induration diameter measured ≥ 10 mm, or if vesiculation or ulceration was observed at the test site, consistent with standard clinical criteria. The IGRA assay quantifies cell-mediated immune responses by measuring Interferon-Gamma (IFN-γ) release stimulated by mycobacterial peptide antigens. An IGRA result was defined as reactive if the following criteria were met: the nil value was ≤ 8.0 IU/mL, the TB antigen minus nil value was ≥0.35 IU/mL, and the TB antigen minus nil value accounted for at least 25 % of the nil value.

For the purpose of this analysis, a participant was considered to have LTBI if either the TST or IGRA result was positive, in line with established epidemiological and clinical conventions for LTBI identification in population-based studies. Additionally, active TB status was defined based on clinical symptoms, radiological findings, and laboratory diagnostic results recorded in the NHANES dataset, in strict accordance with the diagnostic criteria outlined in the Operational Guidelines for NHANES and the diagnostic standards recommended by the Centers for Disease Control and Prevention (CDC). The association between LTBI and active TB was further verified to validate the reliability of LTBI indicators.^18^

### Independent variable

The Dietary Inflammatory Index (DII) was calculated according to the methodology originally developed by Shivappa et al. in 2014.^19^ DII scores quantify the inflammatory potential of an individual’s diet based on the intake of 28 dietary components, including carbohydrates, proteins, fats, vitamins, and minerals.

Dietary intake data were collected via a single 24-hour dietary recall interview administered by trained MEC personnel, which captured participants’ food and beverage consumption in the 24-hours preceding the interview. Individual DII scores were computed by summing the component-specific scores for each of the 28 dietary nutrients, with higher scores indicating a more pro-inflammatory dietary pattern and lower (negative) scores indicating a more anti-inflammatory dietary pattern.^20^^,^^21^

Notably, previous studies have validated the biological relevance of DII scores by demonstrating their correlation with circulating inflammatory biomarkers, including Interleukin (IL)-1β, IL-6, Tumor Necrosis Factor-alpha (TNF-α), C-Reactive Protein (CRP), and White Blood Cell (WBC) counts.^22^, ^23^, ^24^ For the present analysis, DII scores were further categorized into tertiles (low, medium, high inflammatory potential) to facilitate stratified analyses, with each tertile accounting for approximately 33.3 % of the study sample to ensure balanced exposure groups.

### Covariant

Country of birth was treated as a primary confounding variable in this study, categorized as a binary variable (U.S.-born vs. non-U.S.-born) based on self-reported participant data. The authors acknowledge that this binary classification represents a relatively crude measure of nativity-related factors, as it does not capture nuanced differences such as length of residence in the U.S., immigration generation, or country-specific environmental and socioeconomic conditions, which may introduce residual confounding. This limitation is explicitly addressed in subsequent data analysis and discussion sections.

### Statistical analyses

This study was a secondary analysis of publicly available data from the National Health and Nutrition Examination Survey (NHANES). Categorical variables were summarized as frequencies and percentages ( %), while continuous variables were expressed as mean ± Standard Deviation (SD) for normally distributed data or median (interquartile range, IQR) for skewed data, following normality testing. Inter-group differences were compared using one-way analysis of variance (ANOVA) for normally distributed continuous variables, Kruskal-Wallis H test for skewed continuous variables, and chi-square test for categorical variables.

The core analytical objective was to quantify the confounding effect of country of birth on the apparent association between the Dietary Inflammatory Index (DII) and Latent Tuberculosis Infection (LTBI). A sequential adjustment strategy was adopted in logistic regression models to calculate Odds Ratios (ORs) and 95 % Confidence Intervals (95 % CIs), thereby isolating the impact of country of birth:

1. Model 1 (Crude model): No covariates adjusted;
2. Model 2 (Basic adjusted model): Adjusted for demographic factors (age, gender, ethnicity), socioeconomic factors (educational attainment, marital status, Poverty Income Ratio [PIR]), and health-related factors (Body Mass Index [BMI], smoking status, alcohol intake, Hypertension [HPT], Diabetes Mellitus [DM]);
3. Model 3 (Full adjusted model): Further adjusted for country of birth (dichotomized as U.S.-born vs. non-U.S.-born) on the basis of Model 2.

Restricted Cubic Spline (RCS) regression with 4 knots (at the 5th, 35th, 65th, and 95th percentiles of the DII distribution) was separately applied to Model 2 and Model 3, to compare the linear or non-linear dose-response relationship between continuous DII and LTBI risk before and after adjusting for country of birth. The RCS results were used to verify whether the initially observed dose-response trend would disappear after controlling for the confounding variable of country of birth.

Stratified analyses were performed by country of birth (U.S.-born vs. non-U.S.-born) to verify the consistency of the DII-LTBI association across nativity subgroups. Additionally, subgroup analyses were conducted for predefined effect modifiers, including age (< 65 vs. ≥ 65-years), BMI (< 30 vs. ≥ 30 kg/m²), smoking status, HPT, and DM. Likelihood ratio tests were used to assess the interaction between DII and each subgroup variable, with statistical significance set at *p* < 0.05.

DII was categorized into tertiles according to its distribution in the 3890 valid samples: T1 (low inflammatory potential, −4.63 to 0.60), T2 (medium inflammatory potential, 0.60 to 2.49), T3 (high inflammatory potential, 2.49 to 5.47), with each tertile accounting for approximately 33.3 % of the total sample to ensure balanced grouping. Logistic regression analyses were repeated using DII tertiles (with T1 as the reference group) to confirm the robustness of the association results. E-value calculation was conducted to evaluate the potential impact of unmeasured confounding factors on the observed association in Model 2.

Given the secondary data analysis design, no a priori sample size calculation or statistical power analysis was conducted. All statistical analyses were performed using R software version 3.3.2 (The R Foundation for Statistical Computing, Vienna, Austria; accessed on 26 November 2024) and Free Statistics software version 2.0.^25^ Two-tailed tests were used for all analyses, and a p-value < 0.05 was considered statistically significant.

The results from the multivariate logistic regression analysis are presented in Table 1. In the initial crude model (Model 1), DII was marginally inversely associated with LTBI risk (OR = 0.95, 95 % CI 0.90–1.00). This association became statistically significant after adjusting for demographic, socioeconomic, and health-related covariates in Model 2 (OR = 0.93, 95 % CI 0.88–0.98, *p* < 0.05). When DII was analyzed as a categorical variable by tertiles, compared with the T1 group, the T3 group showed a 21 % reduction in LTBI risk in the crude model (OR = 0.79, 95 % CI 0.62–0.99), and this risk reduction was strengthened after covariate adjustment (OR = 0.72, 95 % CI: 0.56–0.93, *p* < 0.05). The E-value for the association observed in Model 2 was 1.29 (CI-bound E-value = 1.00), indicating moderate robustness to unmeasured confounding. Notably, all the above significant associations were attenuated to non-significance after further adjustment for country of birth in Model 3, which directly supports the core hypothesis that country of birth acts as a critical confounder in the DII-LTBI association.

**Table 1:** Multivariate logistic regression models evaluating the association between DII and LTBI.

Variable	NO.	n.event_ %	OR (95 % CI)					
			Crude	p-value	Model 1	p-value	Model 2	p-value
**DII (continuous)**								
DII	3892	496 (12.7)	0.95 (0.90∼1.00)	0.038	0.93 (0.88∼0.98)	0.011	0.96 (0.90∼1.01)	0.126
**DII (classified)**								
T1	1297	180 (13.9)	1 (Ref)		1 (Ref)		1 (Ref)	
T2	1297	170 (13.1)	0.94 (0.75∼1.17)	0.566	0.92 (0.72∼1.17)	0.483	0.96 (0.75∼1.22)	0.725
T3	1298	146 (11.2)	0.79 (0.62∼0.99)	0.044	0.72 (0.56∼0.93)	0.011	0.83 (0.64∼1.09)	0.179
Trend test	3892	496 (12.7)	0.89 (0.79∼1.00)	0.045	0.85 (0.75∼0.96)	0.012	0.91 (0.8∼1.04)	0.184

Multivariate logistic regression adjusted for potential confounders demonstrates a 1-unit increment in the DII reduces the risk of tuberculosis infection by 7 % (95 % CI 0.88, 0.98, *p* = 0.011) (Table 3, Model 1), but this association was no longer significant (OR = 0.96, 95 % CI 0.90‒1.01) after further adjustment for country of birth.

Abbreviations: CI, Confidence ins ratio.

## Results

### Study population

**Fig. 1:**
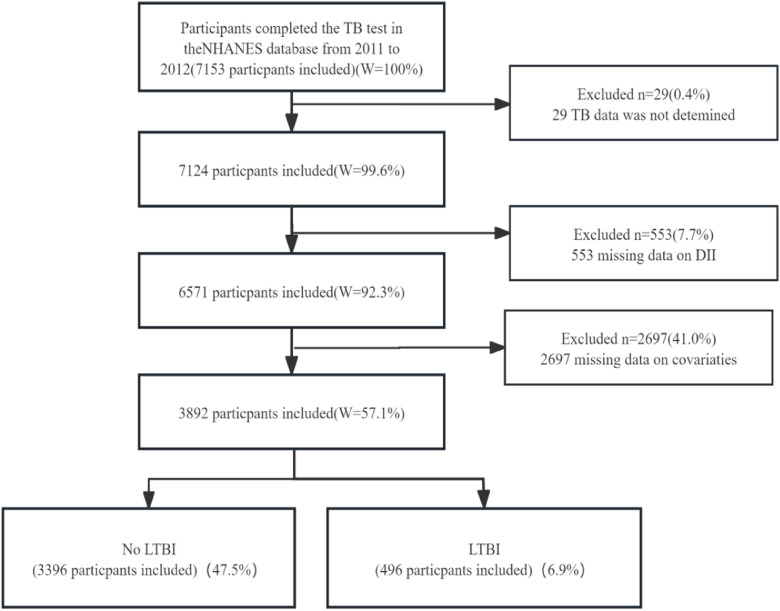
The study’s flow diagram.

A total of 7153 individuals participated in the TB test after the authors excluded those for whom TB data were indeterminate (*n* = 29), those with missing DII data (*n* = 553), and participants lacking information on covariates (*n* = 2697). Consequently, this cross-sectional analysis ultimately included 3892 participants from the NHANES data collected between 2011 and 2012. The detailed process of inclusion and exclusion is depicted in Fig. 1.

### Baseline characteristics

A total of 3892 patients, ranging in age from 20- to 80-years, were included in the analysis. Among them, 496 individuals (12.74 %) were identified as having LTBI, 144 had TST+/IGRA-, 203 had IGRA+/TST-, and 149 had both TST+/IGRA+. The positive concordance rate was 30.04 %. The median (quartiles) of DII in the three groups were 1.7 (0.2, 2.7), 1.1 (−0.3, 2.7), and 1.7 (−0.1, 2.8), respectively. The statistical value was 6.592 with *p* = 0.086, indicating no statistically significant difference in DII among the groups. Table 2 illustrates the overall characteristics of the participants categorized by LTBI status. The mean age of the participants was 48.4 ± 17.8 years, with 1923 (49.4 %) being female. Participants who tested positive for LTBI were predominantly male, older, smokers, alcohol consumers, diagnosed with hypertension or diabetes mellitus, of Other Hispanic or other races backgrounds, married, having attained less than a high school education, and demonstrating a lower Poverty Income Ratio (PIR), a lower DII and the total number of households exceeds 5.

**Table 2:** Population characteristics by categories of LTBI^a^.

Variables	Total (*n* = 3892)	LTBI (*n* = 496)	No-LTBI (*n* = 3396)	p-value
Country of birth, n ( %)				<0.001
USA	2880 (74.0)	2688 (79.2)	192 (38.7)	
Not USA	1010 (26.0)	706 (20.8)	304 (61.3)	
Gender, n ( %)				<0.001
Male	1969 (50.6)	295 (59.5)	1674 (49.3)	
Female	1923 (49.4)	201 (40.5)	1722 (50.7)	
Age	48.4 ± 17.8	53.5 ± 16.1	47.7 ± 17.9	<0.001
BMI	29.0 ± 6.9	28.8 ± 6.8	29.1 ± 7.0	0.451
Smoking, n ( %)				0.011
Yes	795 (20.4)	107 (21.6)	688 (20.3)	
Former	910 (23.4)	139 (28)	771 (22.7)	
Nerver	2187 (56.2)	250 (50.4)	1937 (57)	
Drinking, n ( %)				0.003
No	2919 (75.0)	345 (69.6)	2574 (75.8)	
Yes	973 (25.0)	151 (30.4)	822 (24.2)	
HPT, n ( %)				0.026
Yes	1410 (36.2)	202 (40.7)	1208 (35.6)	
No	2482 (63.8)	294 (59.3)	2188 (64.4)	
DM, n ( %)				<0.001
Yes	486 (12.5)	86 (17.3)	400 (11.8)	
No	3406 (87.5)	410 (82.7)	2996 (88.2)	
Race, n ( %)				<0.001
Mexican American	358 (9.2)	77 (15.5)	281 (8.3)	
non-Hispanic White	1596 (41.0)	74 (14.9)	1522 (44.8)	
non-Hispanic Black	999 (25.7)	131 (26.4)	868 (25.6)	
Other Hispanic or other races	939 (24.1)	214 (43.1)	725 (21.3)	
Marital, n ( %)				0.002
Married or live with partner	2181 (56.0)	310 (62.5)	1871 (55.1)	
Single	1711 (44.0)	186 (37.5)	1525 (44.9)	
Education, n ( %)				<0.001
Less than high school	804 (20.7)	166 (33.5)	638 (18.8)	
High school or above	3088 (79.3)	330 (66.5)	2758 (81.2)	
PIR	2.0 (1.0, 4.1)	1.8 (0.9, 3.6)	2.0 (1.1, 4.2)	0.004
DII	1.6 (0.0, 2.9)	1.5 (−0.1, 2.7)	1.6 (0.1, 3.0)	0.039
Total number of households, n ( %)				<0.001
< 5	3179 (81.7)	370 (74.6)	2809 (82.7)	
≥ 5	713 (18.3)	126 (25.4)	587 (17.3)	

a Data are presented as unweighted number (weighted percentage) for categorical variables and median (quartile) for continuous variables which is not normally distributed.

### Relationship between DII and LTBI

The univariate analysis demonstrated that gender, age, race, education, marital status, drinking, DM, HPT, PIR, Total number of people in the Household, DII were associated with LTBI. However, no significant differences were observed between the groups regarding BMI and smoking (all *p* > 0.05) (Table 3).

**Table 3:** Association of covariates and LTBI.

Variable	OR (95 % CI)	p
**Country of birth**		
USA	1 (Reference)	
Not USA	6.03 (4.94∼7.35)	<0.001
**Gender**		
Male	1 (Reference)	
Female	0.66 (0.55∼0.8)	<0.001
**Age**	1.02 (1.01∼1.02)	<0.001
**Race**		
Mexican American	1 (Reference)	
non-Hispanic White	0.18 (0.13∼0.25)	<0.001
non-Hispanic Black	0.55 (0.4∼0.75)	<0.001
Other Hispanic or other races	1.08 (0.8∼1.45)	0.621
**Education level**		
Less than high school	1 (Reference)	
High school or above	0.46 (0.37∼0.56)	<0.001
**Marital Status**		
Married or live with partner	1 (Reference)	
Single	0.74 (0.61∼0.89)	0.002
**Smoking**		
Current	1 (Reference)	
Former	1.16 (0.88∼1.52)	0.287
Nerver	0.83 (0.65∼1.06)	0.132
**Drinking**		
Yes	1 (Reference)	
No	1.37 (1.11∼1.69)	0.003
**BMI**	0.99 (0.98∼1.01)	0.451
**DM**		
Yes	1 (Reference)	
No	0.64 (0.49∼0.82)	0.001
**HPT**		
Yes	1 (Reference)	
No	0.8 (0.66∼0.97)	0.026
PIR	0.91 (0.86∼0.97)	0.002
**Total number of people in the Household**		
< 5	1.63 (1.31∼2.03)	<0.001
≥ 5	1.63 (1.31∼2.03)	
**DII**	0.95 (0.9∼1)	0.038

CI, Confidence Interval; OR, Odds Ratio.

The results of the multivariate logistic regression analysis for the association between the Dietary Inflammatory Index (DII) and Latent Tuberculosis Infection (LTBI) are summarized in Table 1, with a core focus on quantifying the confounding effect of country of birth.

In the crude model (Model 1, no covariates adjusted), DII showed a marginal inverse association with LTBI risk (OR = 0.95, 95 % CI: 0.90–1.00). After adjusting for demographic factors (age, gender, ethnicity), socioeconomic factors (educational attainment, marital status, Poverty Income Ratio [PIR]), and health-related factors (Body Mass Index [BMI], smoking status, alcohol intake, Hypertension [HPT], Diabetes Mellitus [DM]) in Model 2, this association became statistically significant (OR = 0.93, 95 % CI: 0.88–0.98, *p* < 0.05).

When DII was categorized into three balanced tertiles based on its distribution in the 3890 valid samples ‒ T1 (low inflammatory potential, −4.63 to 0.60), T2 (medium inflammatory potential, 0.60 to 2.49), T3 (high inflammatory potential, 2.49 to 5.47) ‒ logistic regression analyses further validated the association. In the crude model, compared with the T1 group, the T3 group had a 21 % reduction in LTBI risk (OR = 0.79, 95 % CI 0.62–0.99, *p* < 0.05). This risk reduction was strengthened after adjusting for the covariates in Model 2 (OR = 0.72, 95 % CI 0.56–0.93, *p* < 0.05). The E-value for the association observed in Model 2 was 1.29 (CI-bound E-value = 1.00), indicating moderate robustness to unmeasured confounding factors.

Notably, after further adjusting for country of birth (dichotomized as U.S.-born vs. non-U.S.-born) in Model 3, all the aforementioned statistically significant associations were completely attenuated to non-significance. For continuous DII, the adjusted OR shifted to 0.96 (95 % CI 0.90–1.01, *p* > 0.05); for DII tertiles, the T3 group showed no significant difference in LTBI risk compared with the T1 group (OR = 0.83, 95 % CI 0.64–1.09, *p* > 0.05). This finding directly supports the core hypothesis that country of birth acts as a critical confounder in the apparent DII-LTBI association.

**Fig. 2:**
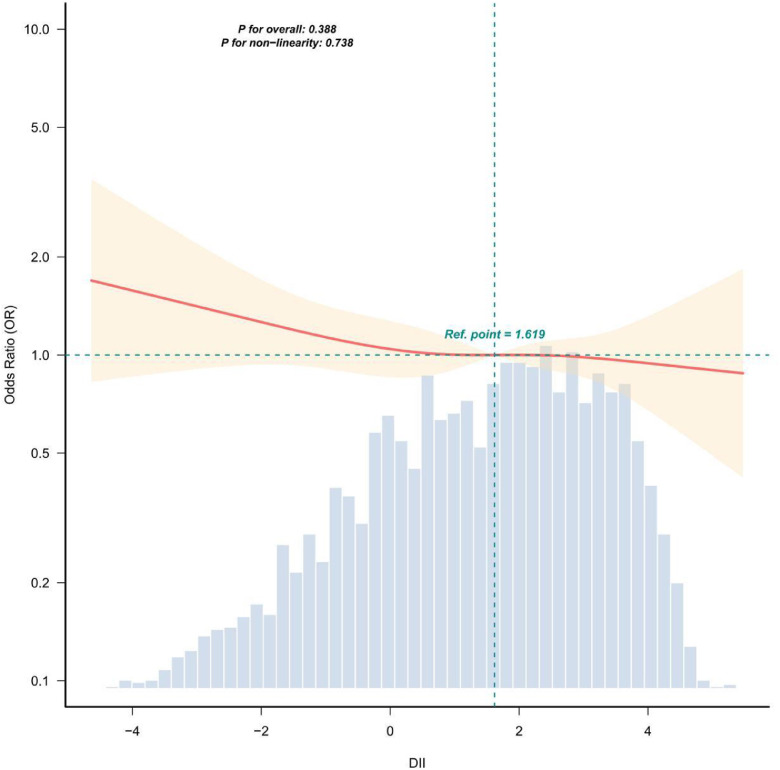
Restricted cubic spline plots for LTBI outcome by DII after covariates adjustment. Adjusted for country of birth, age, gender, race, educational level, smoking, drinking, BMI, PIR, and the total number of households. The dashed lines represent the 95 % Confidence Intervals. Ref. point 1.401 is the median of the x-axis (DII value) and is used to standardize curve comparison.

The adjusted smoothed plots indicate there’s no significance between DII and LTBI (Fig. 2, P for non-linearity = 0.916, with the extreme 0.5 % trimmed for each DII measure).

### Stratified analyses based on additional variables

**Fig. 3:**
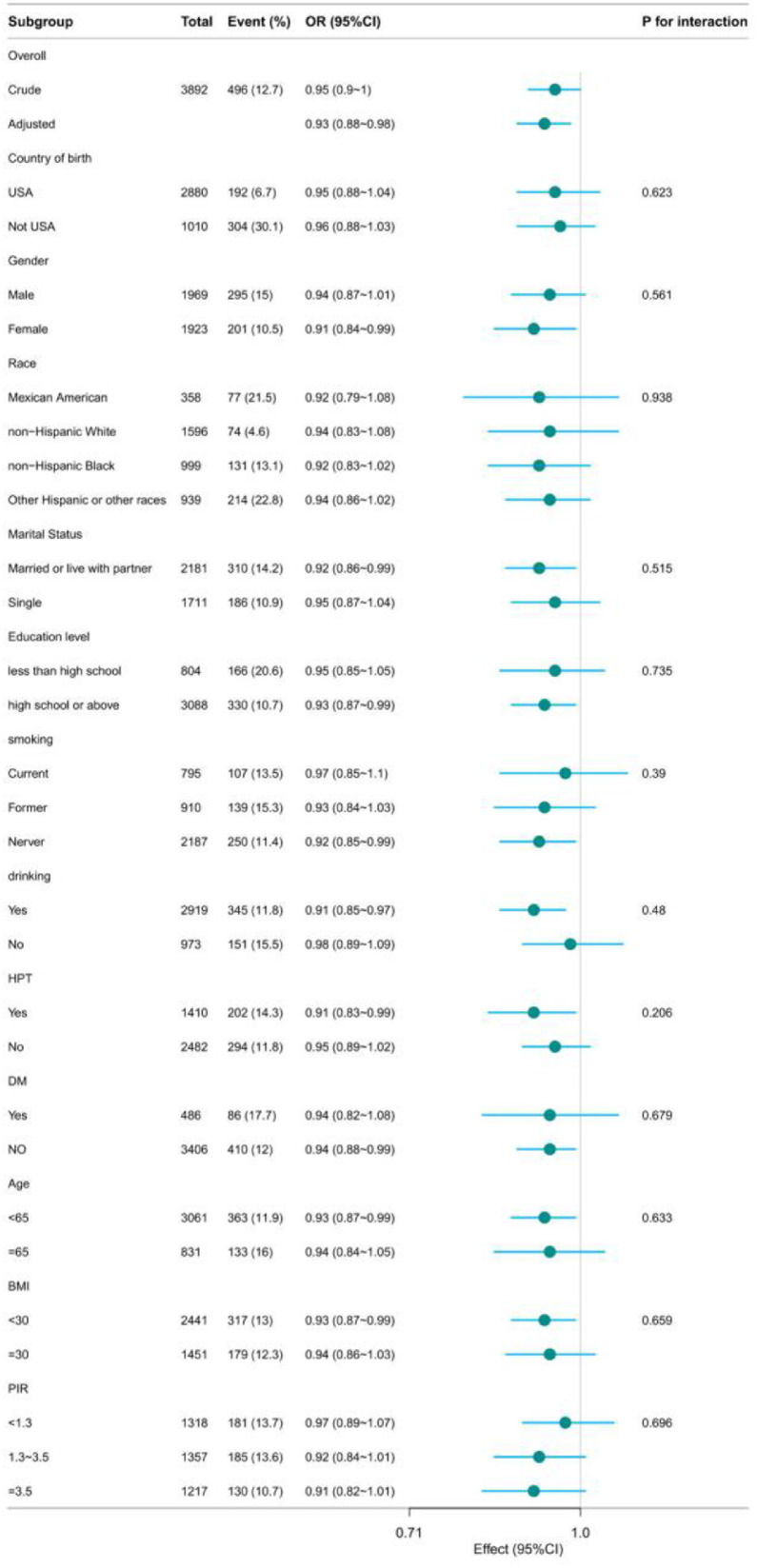
The relationship between DII and LTBI according to basic features. Except for the stratification component itself, each stratification factor was adjusted for all other variables (country of birth, age, gender, race, educational level, smoking, drinking, BMI, PIR, Marital, HPT, DM).

Stratified analyses were conducted across several subgroups to evaluate possible effect modifications concerning the association between DII and LTBI. No notable interactions were identified within any subgroup after stratifying by gender, race, marital status, education level, smoking, drinking, HPT, DM, age, BMI and PIR (Fig. 3).

## Discussion

This study explored the association between the Dietary Inflammatory Index (DII) and Latent Tuberculosis Infection (LTBI) using a cross-sectional analysis. Initial multivariable logistic regression showed a significant negative association between DII and LTBI (OR = 0.93, 95 % CI 0.88–0.98, *p* = 0.011), suggesting that an anti-inflammatory diet may have a protective effect. However, after further adjustment for country of birth, this association was no longer significant (OR = 0.95, 95 % CI 0.88–1.04), and subgroup analyses did not find significant associations in either U.S.-born or non-U.S.-born populations (interaction p-values > 0.05). These results indicate that the initially observed “protective effect” is likely confounded by the confounding variable of country of birth.

Confounding factors are crucial in epidemiological studies. They are related to both exposure factors and outcomes, which can potentially lead to false associations or distort true effects.^26^ In this study, the country of birth may act as a confounding variable through various pathways. First, dietary patterns (reflected in DII) are often influenced by cultural background and geographic origin;^27^^,^^28^ for example, immigrants may retain dietary habits from their country of origin.^29^ Second, the risk of LTBI is highly dependent on tuberculosis endemic areas,^30^ and non-U.S.-born populations may come from high-burden countries,^31^ thus having a higher risk of infection. This dual association makes country of birth a key confounding variable, which, if not controlled, would incorrectly show a negative association between DII and LTBI.

Further analyses corroborate these findings. The subgroup analyses and smooth curve fitting in this study further support the confounding effect of country of birth. No significant associations were found after stratification. Moreover, the dose-response relationship disappeared after adjustment, indicating that country of birth is more likely a confounding variable rather than an effect modifier. This is consistent with existing literature, where socioeconomic status and immigration status are often reported as important confounding variables in studies of diet and infection relationships.^32^^,^^33^

Although this study is based on large sample data from NHANES and controlled for various demographic and clinical variables (such as age, BMI, and smoking history), there are still limitations. The cross-sectional design cannot infer causality; however, there may be unmeasured confounding variables (such as genetic background or specific dietary components). Additionally, the data rely on self-reported information, which may introduce recall bias.

From a public health perspective, these findings emphasize that future research must systematically assess and adjust for potential confounding variables such as country of birth when exploring the association between diet and infection. In clinical practice, recommendations for anti-inflammatory diets should consider individual backgrounds to avoid overinterpreting preliminary associations. Future research directions include prospective cohort or experimental studies to verify the true role of DII in tuberculosis prevention and integrate multidimensional confounding variable analyses.

## Conclusions

This study demonstrates that the initially observed inverse association between the Dietary Inflammatory Index (DII) and Latent Tuberculosis Infection (LTBI) was not sustained after controlling for the confounding variable of country of birth. These findings highlight that country of birth is a critical confounding factor in studies examining the relationship between diet and infection. Future epidemiological investigations should systematically adjust for such factors to accurately evaluate the potential role of dietary interventions in tuberculosis prevention.

## Ethics committee study protocol number

This research protocol is based on the continuation protocol (Protocol #2005–06) approved by the Institutional Review Board of the National Center for Health Statistics (NCHS). All research subjects signed written informed consent forms before being enrolled in the original survey. Secondary analysis of de-identified NHANES data (2011‒2012 cycle) does not require additional institutional review board approval. NHANES data can be publicly accessed through the official website, and data extraction was completed on November 26, 2024.

## Data availability

Publicly available datasets are available online for this study. The repository/repositories name and accession numbers are available online at https://wwwn.cdc.gov/nchs/nhanes/default.aspx (accessed on 26 November 2024).

## Authors’ contributions

Jingbo Jia: Collected, analyzed and interpreted the data and results, and drafted the manuscript.

Yuanyuan Liu: Proposed the concept and design of the study and revised the manuscript for critical intellectual content.

Hua Zhang: Performed data cleaning of this paper, guidance on statistical analysis and revision of the manuscript.

Jianrui Pi: Was responsible for implementing the study and collecting the data.

Chao Wang: Is responsible for collecting the data and analyzing the data.

Shiqi Dong: Was responsible for implementing the study and collecting the data.

Tong Zhang: Was responsible for implementing the study and drafting the article.

Wanjie Yang: Revised manuscript for critical intellectual content.

All authors had access to the data. All authors read and approved the final manuscript.

## Funding

This study was funded by Tianjin Key Medical Discipline Construction Project (Grant no TJYXZDXK-3–018B).

Ethics approval was obtained from the NCHS Ethics Review Committee, and participants provided written informed consent. The secondary analysis did not require additional institutional review board approval.

## Declaration of competing interest

The authors declare no conflicts of interest.
